# After all, what is the proper use of Kappa statistics in oral health surveys? What don’t manuals tell us?

**DOI:** 10.1590/1807-3107bor-2025.vol39.102

**Published:** 2025-10-10

**Authors:** Andréa Videira ASSAF, Renato Pereira da Silva, Fábio Luiz Mialhe, Antonio Carlos Pereira

**Affiliations:** (a) Universidade Federal Fluminense – UFF, Health Institute of Nova Friburgo, Nova Friburgo, RJ, Brazil.; (b) Universidade Federal de Viçosa – UFV, Department of Nutrition and Health, Viçosa, MG, Brazil.; (c) Universidade Estadual de Campinas – UNICAMP, Piracicaba Dental School, Department of Community Dentistry, Piracicaba, SP, Brazil.

**Keywords:** Dental Caries, Epidemiology, Data Interpretation, Statistical, Reproducibility of Results

## Abstract

The aim of this study was to elucidate, unclear points of the “Oral Health Survey: basic methods”, of the World Health Organization (WHO), relative to reproducibility (encompassed reliability and agreement) issues during examiners’ calibration. Thus, Kappa statistics and percent agreement were calculated for a sample of 10 12-year-old schoolchildren examined by 1 gold standard examiner and 5 dentists from Nova Friburgo, RJ, Brazil, in 2018, under the WHO and SB Brasil 2010 Project settings. Weighted Kappa was used to measure reliability between 2 examiners, and Fleiss’ Kappa for 5 examiners. Tooth-to-tooth reliability was also assessed. The results showed that, although the choice of different settings invariably produced different reliability and agreement values, this approach was feasible, coherent and even desirable depending on the purpose of an epidemiological survey conducted. Kappa values were slightly lower in the SB Brasil 2010 Project setting. The results for tooth-to-tooth reliability, in turn, allowed identification of teeth (in this sample, teeth 17, 23, 27, 34, 37, 44, 45, and 47) for which additional examiner calibrations would be necessary. It is concluded that providing additional information for inclusion in the WHO manual, such as the possibility of varying the setting, adopting the tooth-by-tooth unit, and selecting the correct type of Kappa statistic depending on the number of examiners, within a multilevel calibration proposal, may result in more reliable results during the calibration stage.

## Introduction

Oral health surveys are the basis for surveillance of disease patterns. The data obtained from these surveys are useful for action planning and decision-making by dentists and managers, especially from a public health perspective.^1^ Nowadays, the global pattern of dental caries development – characterized by its general decline across different age groups and countries,^2-5^subsequent polarization in specific age groups,^6-8^ and by the concentration of caries lesions in molar teeth^9,10^ – makes its diagnosis a more complex task. Moreover, it is common for differences in examiners’ judgments to arise, when they assess the oral health status of individuals, and these differences may be related to their physical and emotional states,^11^ as well as their previous knowledge and experience with dental caries diagnosis.^12^

In view of the global pattern of dental caries, various diagnostic criteria and thresholds (e.g., WHO diagnostic criteria, ICDAS, CAST, PUFA, etc.) are in use at present. Each criterion or threshold has a specific indication.^13-16^ Irrespective of the criterion or threshold adopted, the entire calibration process is designed to prepare examiners for the collection of all pertinent data. The primary objective of this process is to ensure consistency, i.e., a uniform interpretation, understanding, and application of the criteria for diseases and conditions to be observed and recorded, thereby minimizing variations among different examiners.^11,17^ This process is conducted under clinical guidelines such as “Oral Health Surveys: basic methods”^11^ or similar manuals.^13,17^These manuals also present clinical specificities and health risks for each age group.^11,13,17^ However, the aims of such manuals are sometimes difficult to achieve in the field.^18^ A superficial approach to certain methodological aspects in these manuals contributes to this situation.^1^ Thus, several studies^1,15,16,19-21^ have been published with the aim of contributing to improving the methodology recommended in “Oral Health Surveys: basic methods”.^11^ These in depth studies address the methodological aspects that directly impact the reproducibility and accuracy of exams performed in epidemiological surveys.^1,15,16,19-21^

Despite their similar appearance, and the fact that they are often used as interchangeable terms, “reproducibility”, “reliability” and “agreement” are concepts that relate to different, yet complementary, items of information.^22-24^ Reproducibility refers to the degree to which repeated measurements on the same subjects produce the same results.^25^ Therefore, reproducibility can be understood as an umbrella concept that encompasses the terms reliability and agreement.^24,25^

In this context, reliability and agreement analyses are important tools in the calibration process.^22,24,26^ It is important to remember that the calibration process includes several theoretical-practical exercises and calibration itself, together with the respective reliability and agreement analyses.^11^ Results of reliability and agreement studies indicate the amount of error inherent in diagnostic tests or other measurements in medical sciences. The recommendation to use percentage agreement and Kappa combined in medical studies^22,23^ is fully applicable and desirable in Dentistry as well.^11^ But do manuals for epidemiological surveys in Dentistry provide all the information regarding reliability and agreement that we need? This study aim is to answer that question.

## Methods

Secondary data from the calibration process for the municipal oral health survey of Nova Friburgo, Rio de Janeiro, Brazil, in 2018, were used to illustrate topics to answer the above-mentioned question. The project of this survey was approved by the Research Ethics Committee from the Health Institute of Nova Friburgo, Rio de Janeiro, Brazil, protocol n^o^. 2.402.679 – CAAE: 76415817.2.0000.5626.

The Nova Friburgo oral health survey investigated the dental condition of 12-year-old schoolchildren from a public school. In this survey, the SB Brasil 2010 Project examiners’ calibration manual^17^ and its electronic spreadsheet were adopted for calculating reproducibility. This spreadsheet was configured to calculate reproducibility between 1 gold standard examiner and up to 5 dentists.^17^ Its final calibration exercise was chosen to answer the guiding question of the present study. This calibration exercise was performed by 1 gold standard examiner and 5 dentists from the Unified Health System (SUS), who examined a sample of 10 12-year-old schoolchildren as prescribed in the SB Brasil 2010 Project examiner’s calibration manual.^17^ Despite not being necessary to use a gold standard examiner in reproducibility studies, his/her presence provides the calibration process with greater safety and quality.^18^

Kappa statistics and percentage agreement were calculated according to “Oral Health Surveys: basic methods”, 5^th^ edition,^11^ and then according to the examiner calibration manual of the “SB Brasil 2010 Project”.^17^ The first mentioned document is adopted worldwide, while the second is an adaptation of its 4^th^ edition, used as the standard for oral health surveys in Brazil. In the first document, there is no specification of which clinical conditions to use for calculating reliability and agreement, thus all codes were considered in this calculation.^11^ According to the Brazilian manual, Kappa statistics are calculated considering codes 0 - sound, 1 - caries, 2 – filled, with caries, 3 – filled, no caries, and 4 – missing due to caries (the most relevant codes for public health planning and surveillance) from the WHO diagnostic criteria.^17^

Both reliability and agreement were analyzed using the software R version 4.3.2 (2023-10-31 ucrt) -- “Eye Holes”, The R Foundation for Statistical Computing.

Weighted Cohen’s Kappa (κ) was used to determine the reliability between the gold standard examiner and each dentist, considering categorical data in the sample.^27^ This type of Kappa is indicated for measuring reliability between 2 examiners only. To calculate reliability among 3 or more examiners simultaneously, Fleiss’ Kappa is the method of choice.^23^The reliability and agreement for each tooth were also analyzed. This methodology allows for a detailed description of reliability calculations among examiners. Fleiss’ Kappa (κ) was used for this purpose as well.

The Kappa values obtained were interpreted according to the classifications of McHugh^23^and Landis and Koch.^28^

## Results

Alternating between “considering all codes from the WHO diagnostic criteria”^11^ and “only codes of relevance to public health”^17^ in calculating reliability and agreement produced different results among the pairs of examiners (Table 1).

**Table 1:** Weighted Cohen’s Kappa by pairs of examiners, according to WHO and SB Brasil 2010 manuals Nova Friburgo, RJ, Brazil, 2018.

Pairs of examiners	WHO			SB Brasil 2010		
	Kappa		% agreement	Kappa		% agreement
	κ [95%CI]	p-value		κ [95%CI]	p-value	
Std*Ex1	0.96	< 0.001	94.1	0.83	< 0.001	94.8
	[0.93–0.99]			[0.70–0.95]		
Std*Ex2	0.97	< 0.001	97.8	0.91	< 0.001	98.1
	[0.94–1.00]			[0.81–1.00]		
Std*Ex3	0.97	< 0.001	95.6	0.90	< 0.001	95.9
	[0.93–1.00]			[0.81–0.99]		
Std*Ex4	0.97	< 0.001	94.1	0.87	< 0.001	94.4
	[0.95–1.00]			[0.76–0.97]		
Std*Ex5	0.97	< 0.001	95.3	0.86	< 0.001	95.2
	[0.95–1.00]			[0.75–0.96]		

As seen in Table 1, all Kappa values from the SB Brasil 2010 setting were classified as “excellent” (κ = 0.81–1.00), according to Landis and Koch.^28^However, considering McHugh’s classification,^23^ only pairs of examiners 2 and 3 presented an “almost perfect” (κ > 0.90) strength of agreement for this setting. The other pairs, in the SB Brasil 2010 setting, presented a “strong” (κ = 0.80–0.90) strength of agreement.

Reliability and agreement for all the examiners together are presented in Table 2. Changing settings from WHO to SB Brasil 2010 resulted in reduced reliability in this sample. The reliability decreased from “strong/excellent” to “moderate/substantial” according to McHugh^23^and Landis and Koch^28^classifications. However, considering each code, an improvement in reliability for codes 3 and 4 was observed. In both settings, code 2 requires special attention during calibration. Code 5 (WHO setting) also deserves special attention.

**Table 2:** Fleiss’ Kappa for the sample, Nova Friburgo, RJ, Brazil, 2018.

Sample	WHO			SB Brasil 2010		
	Kappa		% agreement	Kappa		% agreement
	κ [CI 95%]	p-value		κ [CI 95%]	p-value	
Sample	0.868	0.000	86.2	0.757	0.000	86.8
	[0.8494268 – 0.8875365]			[0.7346374 – 0.7801726]		
Codes						
0	0.899	0.000		0.805	0.000	
1	0.643	0.000		0.639	0.000	
2	0.064	0.000		0.063	0.000	
3	0.781	0.000		0.807	0.000	
4	0.799	0.000		0.908	0.000	
5	-0.001	0.971				
6	0.770	0.000				
8	0.972	0.000				
A	0.857	0.000				
B	0.799	0.000				

The tooth-by-tooth reliability (according to Landis and Koch^28^classification) revealed that teeth 17, 23, 24, 27, 34, 37, 44, 45 and 47, require additional calibration sessions before examiners began with collection of the main data for the dental caries survey (Table 3). Despite a percentage of 100% agreement among the examiners for the 3^rd^ molars and lower anterior teeth, the Kappa statistic could not be calculated. This occurred because these groups of teeth presented a single clinical condition: all 3^rd^ molars examined received code 8 while all lower anterior teeth received code 0. The calculation of Kappa is only possible when there are at least 2 clinical conditions (2 codes or categories) present in the teeth examined (e.g., presence of 3^rd^ molars with caries (code 1) and without caries (code 0) in the sample examined), thus allowing the construction of a 2x2 contingency table.^23,28^

**Table 3:** Fleiss’ Kappa (κ) and percentage agreement (%) for each tooth from the sample, Nova Friburgo, RJ, Brazil, 2018.

Upper jaw	18	17	16	15	14	13	12	11	21	22	23	24	25	26	27	28
**κ**	-	0.56	0.82	0.78	0.78	0.69	1.00	0.84	0.61	0.84	-0.02	0.17	0.90	0.71	0.49	-
**%**	100	70	70	90	90	80	100	90	90	90	90	90	90	50	60	100
**Lower jaw**	**48**	**47**	**46**	**45**	**44**	**43**	**42**	**41**	**31**	**32**	**33**	**34**	**35**	**36**	**37**	**38**
**κ**	-	0.52	0.87	-0.02	0.17	-	-	-	-	-	-	-0.02	0.82	0.80	0.56	-
**%**	100	60	80	90	90	100	100	100	100	100	100	90	90	70	40	100

## Discussion

Although e a considerable number of scientific articles have addressed the reliability of clinical or epidemiological examinations and laboratory tests in Dentistry, there is little detailed information about the selection and use of statistical tests to measure reliability. The main strength and contribution of this study is that it has provided the explanation of methodological and statistical points that would improve reliability among examiner, and., therefore, the quality of data from oral health surveys. So, do manuals for epidemiological surveys in Dentistry provide all the information regarding reliability and agreement that we need? The direct answer is “no”. Then, What items of information do the manuals fail to give us?

## Methods

Initially, it is essential for the entire team involved – coordinators, recording and organizing clerks, and examiners – to undertake a thorough reading of the “Oral Health Survey: basic methods”.^11^ This reading minimizes the occurrence of mistakes (often irreparable) that can negatively impact the quality of the oral health survey data. Although there are more sensitive thresholds and diagnostic criteria for dental caries, the simplicity and ease of use of the WHO criteria, under the DMF index, favor their adoption as a global standard in epidemiological studies of oral health.^1,16^ Knowing what the calibration process entails and recognizing its importance is crucial for the success of the intended oral health epidemiological survey.^1,11^ However, in the WHO manual, information about the calibration process is vague, superficial, and insufficient. Even in its 5^th^ edition, It does not offer further details about this critical stage,^1,19^.^11^ Reliability data are generally poorly described in epidemiological surveys.^18^

The term “calibration” in oral epidemiology refers to the “*process that aims to establish uniform standards for epidemiological examination in oral health and determines acceptable parameters of internal and external consistency for examiners”* (SB Brasil 2010 Project manual,^17^ p. 6). Calibration is the examiners’ first contact with the population to be examined, making it a crucial aspect of oral health survey planning. It allows for better-defined criteria for exams to be established, and for examiners to have a better understanding of these criteria.^18,19^ Therefore, in addition to discussing the distribution of disease prevalence, calibration sample size^20,^ and the inclusion of teeth without inter-examiner clinical variations in the reliability calculation,^21^ it is important to define which WHO codes to include in the reliability calculation, and how to proceed when more than 2 examiners simultaneously participate in the calibration. Noteworthy is the fact that these topics are not described in the “Oral Health Surveys: basic methods”.^11^

Kappa statistics and percentage agreement are two common ways that have been recommended for assessing intra- and inter-examiner reliability and agreement in dental caries surveys.^11,23,26,29^ As Kappa is a chance-corrected measure, its values tend to be lower than the general percentage of agreement values.^19,27,29^ It is necessary to differentiate between reliability and agreement, in order to select statistical approaches and enable professionals to make adequate interpretations.^23^ However, this differentiation is not addressed in either of the manuals adopted in this study.^11,17^ While reliability can be defined as the ability of a measurement to differentiate between scores of the same subjects (e.g., by different examiners or at different times) relative to the total variability of all scores in the sample, the agreement can be defined as the degree to which scores or ratings are identical.^22^ In other words, reliability is the ability of a measure to be applied twice upon the same examiners to produce the same results on both occasions, while agreement is the capacity of a test to replicate the same ordering between examiners when measured twice.^30^ While reliability is highly dependent on the variability within the sample studied, agreement tends to be more stable in studies that address different population samples.^25^ Reliability and agreement are two distinct concepts that assess different aspects of a diagnostic test. While reliability verifies the consistency of the results of the diagnostic test over time or conditions, agreement assesses whether the results obtained accurately reflect the real variability among the subjects in the sample studied.^24^ Thus, although this study focused on aspects of measuring reliability based on Kappa calculation, both concepts - reliability and agreement - are important and complementary in providing information relative to measurement quality.^22-24^

### Statistical issues

Despite criticism relative to its suitability in determining intra- and inter-examiner reliability,^26^the Kappa statistics continues to be the method of choice for determining reliability in epidemiological studies in oral health conducted under the methodology outlined in the WHO mnaual^11,19^or its derivatives, such as the examiner calibration manual of the SB Brasil 2010 Project.^17^ However, what is not said about the Kappa in dental caries surveys manuals?

No mention is made of which type of Kappa (whether simple or weighted, whether Cohen or Fleiss) in the “Oral Health Surveys: basic methods”.^11^ Details about Cohen’s Kappa (simple and weighted) are provided in the SB Brasil 2010 Project examiners manual.^17^ This manual even mentions a minimum weighted Kappa value of 0.65 to continue the epidemiological survey. The Landis and Koch^28^ classification of strength of agreement is adopted in both manuals.^11,17^ However, McHugh’s^23^ classification seems to be more appropriate today.

As seen in Table 1, the use of weighted Cohen’s Kappa is useful in determining reliability for gold standard-examiner pairs. Low Kappa values for pairs indicate the need for more calibration sessions or, ultimately, the exclusion of examiners. The “Oral Health Surveys: basic methods” provides a brief explanation of this.^11^ The consideration of all clinical codes in the reliability calculation is implied in this manual.^11^When considering only codes of interest to public health^17^ for this sample, the values found for Kappa were slightly lower (Table 1). However, we believe that this is the most appropriate way to calculate reproducibility in population-based dental caries surveys intended for epidemiological surveillance and public health planning. Therefore, this would be the first proposal for making a contribution to the improvement of Oral Health surveys: basic methods.^11^

Another useful piece of information, although not mentioned in the WHO manual,^11^ is the possibility of calculating tooth-by-tooth reliability.^19^ This method works as a refinement of calibration. Divergent results observed in each tooth would not be diluted in the set of all teeth (mostly “sound/WHO code 0” nowadays) analyzed together, as shown in Table 3. Thus, the false impression of good agreement would be mitigated under this way of calculating Kappa.^19,31^ Although unusual, “tooth-by-tooth” reliability is useful for obtaining a more accurate level of diagnosis. Teeth whose caries diagnosis is more complex will present lower reliability values, enabling specific calibration sessions for examiners for those teeth.^19,31^ The effect of considering all WHO codes or only those relevant to public health is irrelevant in calculating tooth-by-tooth reliability. For an even more accurate level of dental caries diagnosis, its variable “surface-to-surface for each tooth” unit can be implemented in examiners’ calibration sessions.^18,31^ In this study, the calculation of tooth-by-tooth reliability was carried out considering more than 2 examiners, so Fleiss’s Kappa was chosen. The tooth-by-tooth^19^ (or surface-to-surface when necessary) reliability is another proposal for improving the WHO^11^ and similar manuals.

In light of the results found, we believe that better reliability results can be achieved by carrying out a multilevel calibration^18^, considering the number of examiners, age range/type of dentition studied and thresholds and diagnostic criteria adopted. Below, we present a proposal for multilevel calibration, considering more than 2 examiners, permanent dentition, and WHO diagnostic criteria (Figure).

**Figure:**
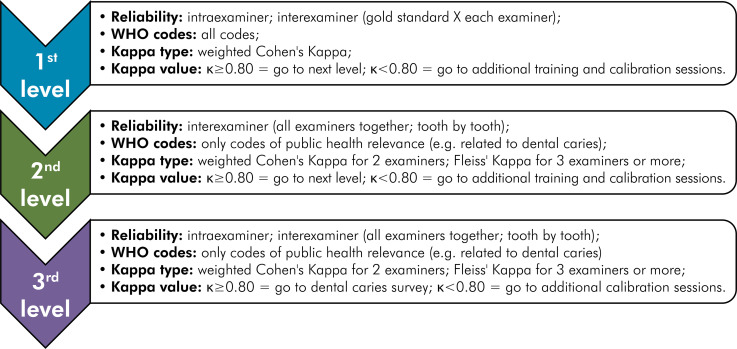
Multilevel calibration for dental caries epidemiological surveys.

Guidance on how to proceed when faced with high variability in calibration is superficial in the WHO manual.^11^ No information about the number of calibration sessions that an examiner with low reproducibility should undergo is provided. Given the logistics and deadlines involved in conducting a national oral health survey, we suggest that an examiner who demonstrates low intra- and inter-examiner (relative to the gold standard examiner) Kappa values in the first calibration session should undergo 1 additional session. If examiners achieve an adequate level of reliability in this session, they can join the team of examiners conducting the oral health survey. Otherwise, they should be replaced by new examiners who will go through the entire calibration process.

The same guidance regarding the number of examiners and type of Kappa in calibration sessions should be implemented in duplicate examinations on 5-10% of the sample (no fewer than 25 subjects) in the actual survey, as proposed in the WHO manual.^11^

A possible approach to simplify the guidelines in the WHO manual^11^ as regards he definition of disease prevalence in examiner calibration samples would be to equalize the number of subjects with high, medium, and low prevalence of dental caries. This type of measure would be sufficient to ensure examiner reliability for a given population under study.^18,19^This would be another proposal for improving the WHO manual^11^ and similar ones.

## Conclusion

Definitely the answer to the question “do manuals for epidemiological surveys in Dentistry provide all the necessary information regarding reliability and agreement that we need?” is: No! Although the “Oral Health Surveys: basic methods”^11^ is an important document that has standardizes oral health surveys internationally, additional information from studies such as the present study adds robustness to its methods, especially relative to the reliability of its examinations. By improving inter-examiner reliability, the trustworthiness of examinations in oral health epidemiological surveys will also be enhanced. Therefore, a more robust calibration process, bridging the gaps present in the WHO manual^11^, such as the type proposed above, is needed to provide more reliable results at the calibration stage. Conversely, we consider that the methodological aspects of epidemiological surveys need to be revised frequently due to the dynamic nature of the profile of dental caries development around the world.^1,19^Therefore, additional methodological studies, considering different prevalence of dental caries,^20^ diagnostic thresholds and criteria,^14,15,16^ units of measurement and of reproducibility,^19,21^ among other factors,^1^ are always welcome.

## Data Availability

The contents underlying the research text are contained in the manuscript.
